# Medical Notes on China

**Published:** 1846-07

**Authors:** 


MEDICAL NOTES ON CHINA. By *John Wilson*, M.D. F.R.S. &c. Inspector of Naval Hospitals and Fleets. 8vo. pp. 267. London 1846. Churchill.

AT the beginning of the year 1842, the Minden, a two-decker, was fitted up as a hospital-ship, and sent out to China, in consequence of our continued hostilities with that country, under the command of Capt. Quin. The medical department was entrusted to the superintendence of our author. Everything appears to have been provided by the Admiralty on the most liberal scale. The limit to the supply of medicines, medical necessaries, comforts and nutriments, including wine, ale, &c., was that of demand only, and this was left to the discretion of Dr. Wilson. It is altogether very pleasing to read of the excellent arrangements that were made to render the vessel a complete and efficient moveable hospital in every respect.

She arrived at Chusan on the 15th of August. From about the time of reaching the Cape of Good Hope, there had been a tendency on board to wounds and other injuries assuming the characters of sloughing ulceration. This was probably owing to the operation of a partially vitiated state of atmosphere on board, the result of crowding and inefficient ventilation, with which the unvarying diet of the crew, and also high temperature of the weather, may have co-operated; for we find that "on the long passage upwards of five hundred men were located in the main deck, which could not be so well ventilated as that portion of ships of war generally are, on account of the whole deck being planked over to protect the men from wet; but while that object was attained, the not less essential one of efficient ventilation was necessarily interfered with. Reid's ventilating apparatus was diligently worked, but as it was constructed for the hospital portion of the ship, the lower and orlop decks, it could exert no direct influence on the main-deck. At the same time, those decks, the lower and orlop, were filled with stores for the fleet, including salted provisions, from which there might be emanations prejudicial to health, and contributing to the vitiated atmosphere which excited the sloughing ulcer. It is right to state that the ship was rendered as clean as her crowded state would permit, and that the pump-well was kept dry and well-aired, through the medium of a tube connected with the ventilating machine."

Although the temperature at Chusan was not very high—the range being from about 76 to 84—upon the ship's arrival in the Chinese seas, much languor and depression were experienced by most of the crew. Diarrhœa and bronchial affections were found to be the prevailing diseases in the crews of the Thalia and Wanderer, the only two vessels lying in the harbour. In the North Star, then off the mouth of the Woosung River, a few cases of malignant Cholera had occurred: several of these proved rapidly fatal. It may be worth noticing that, in some of these cases, there was no purging, and but very little vomiting; yet the collapse was as great as if there had been profuse evacuations upwards and downwards.

During the month of September, we learn that "the principal form of diseased action in this harbour (Chusan) continued to be febrile, with tendency to, if not in every instance, complete development of periodic movements, and affections of the mucous surfaces, either of the alimentary or respiratory organs. Besides diarrhœal complaints, there have been many cases characterized by frequent vomiting and purging of watery fluid, with rather severe tormina occasionally, and feelings of faintness, constituting a mild modification of common atmospheric cholera."

All the prevailing diseases exhibited the characters, more or less distinctly, of an adynamic or asthenic type—weak vascular re-action, and general debility. Hence the use of lowering remedies, especially blood-letting, was not well borne. In some cases there was a sudden and unaccountable sinking of the vital powers.

In the beginning of October, there were received on board the Minden, fifty soldiers of the 98th Regiment, which had suffered terribly from intermittent fever, dysentery, &c., while engaged in military operations upon the coast of the mainland in the Yang-tse-Kiang. This regiment had entered the river, little more than three months before, upwards of 800 strong; and now but 70 rank and file were capable of performing even the lightest duty: there was not one healthy man among them. Dr. Wilson very properly enquires, how it came to pass that this regiment should have suffered so much more than any other, seeing that all the troops were alike exposed to the miasmatic influence of the climate. He is inclined to attribute a good deal to the circumstance of the existing state of the health of the men at the period of their arrival in the Chinese seas. The 98th left England in December 1841, on board the Belleisle, of 72 guns. This ship took out no fewer than 1300 persons in all, including the crew, and she was upwards of six months upon her voyage. Not to allude to the necessarily imperfect ventilation of the decks, when so many were collected together, and to the consequent depravation of the air, it is to be remembered that very few fresh meals could be procured for such a company during the whole of that period. It says much indeed for the excellence of the present arrangements on board our ships of war, that neither scurvy nor any other epidemic broke out among this large assemblage of human beings crowded together in so small a compass. But, as Dr. Wilson very justly remarks, though regulations can accomplish much, they are not omnipotent; they may modify and counteract for a time the hurtful influence of certain agencies; but they cannot eventually resist the injurious effects of crowding, accumulation of animal exhalations, imperfect ventilation, uniform diet, and monotonous manner of life. He therefore most reasonably argues that these noxious influences, "although they did not excite disease on the voyage, yet, by the constitutional debility and deterioration which they induced, they rendered the body highly susceptible to the action of the causes of endemic disease on the shores of the Yang-tse-Kiang, to which the soldiers were exposed on their first landing;" and he then throws out a useful suggestion that deserves to be thought of by officers under like circumstances:—

"It may be concluded that, if the men, at the completion of the voyage, could have been landed for a while, when, with fresh air, moderate exercise and fresh provisions, they might have conjoined healthful amusements, they would at the same time have gained constitutional vigour, and lost susceptibility to disease before entering on active field operations, and thus have escaped the excess of disease and destruction, which all but annihilated their efficiency on first disembarking in China. It is stated, as strongly confirmatory of the above opinion, and is a very important fact, that the men longest quartered on the orlop deck, where ventilation was particularly defective, suffered in a much higher ratio than the others on landing." P. 32.

We need scarcely say that many of the poor fellows of the 98th, the victims of the malarious poison on the Chinese coast, sunk soon after their admission into the hospital-ship. The following is Dr. Wilson's account of the post-mortem appearances generally found in the bodies of the dysenteric patients.

"The examinations have presented in all cases, though in different degrees, extensive disorganization of parts, the seat and general character of the destructive change being remarkably alike in the whole. Ulceration has been all but universal, and obviously in most instances of long standing, the ulcers being scattered more or less thickly over the whole of the rectum and colon, of various forms and superficial dimensions, generally circular, and from the diameter of a split-pea to that of a shilling or more, the surface being purulent, hæmorrhagic, ragged, or clean and smooth, like the effect of excision; of various depths, sometimes destroying the mucous tissue only, sometimes the muscular also, being generally arrested by the peritoneal, but occasionally perforating it. In most cases, the rectum has been much thickened and indurated, in many assuming a semi-cartilaginous character; in other cases, it and the colon have been attenuated in some places while they were thickened in others; and in both, at spots free from absolute ulceration, the mucous lining has been loose and lacerable, or abraded. Frequently there has been serous infiltration of the peritoneal covering, the mucous lining having a leaden hue, through which light-coloured spots were sometimes interspersed, giving it then the appearance of dark marble; occasionally, it has had a purple colour, with a rough granular surface, resembling the exterior of a mulberry; and it has not unfrequently happened that the whole tube has been so much softened, broken-down, and disorganized, as to tear on the slightest touch, or fall to pieces on being lifted. With scarce an exception, the organic lesions have been limited by the ileo-cœcal valve, the small intestines and stomach presenting a perfectly healthy appearance, although venous congestion and small spots, denoting increased vascular action, have been found occasionally. When it is said that the small intestines and stomach presented a perfectly healthy appearance, it should be added that portions of the former, generally the distal extremity of the ilium, were often, in cases of long duration, much attenuated, and pale, and diaphonous, like a prepared colourless membrane." P. 57.

In two cases only, was the structure of the Liver at all affected. Indeed, with the exception of the great intestines alone, all the organs and tissues of the body were, in a vast majority of instances, perfectly healthy. The amount of disorganization in the great gut, that is sometimes compatible with the prolongation of life, is truly wonderful. It was often observed that the patients shortly before death seemed to be somewhat better: after some slight exertion, such as emptying the bowels, gargling the mouth, or so forth, they became faint, and soon expired. In some of these cases, there had been a profuse salivation (not the effect of mercury) accompanied with ulceration of the gums, œdema of the cheeks, &c.: this was always a bad symptom.

The number of cases of Sloughing Ulcer received on board the Minden, for some months after her arrival at Chusan, was considerable. In some, the ulcerative destruction of parts was very deep and extensive. In one case, whose particulars are related, the ulcers commenced on the dorsum of both feet and over the lower ends of the tibia and fibula.

" When death took place, the right foot was in a state closely approaching that of a perfect skeleton, the metatarsal, and great part of the tarsal bones, being not only exposed, but as completely denuded, and cleared of every incumbent tissue, as if they had been carefully dissected. The articulating structures between the metatarsal and phalangeal and tarsal bones respectively, were also completely destroyed; so that it was necessary, some time before death, when dressing the foot, to support it with care at the extremities, to prevent the metatarsal dropping from its tarsal portion. The ulcer on the left leg, at death, embraced three-fourths of its circumference at its lower third, and extended upwards three inches, leaving the tibia and fibula bare for two inches." P. 73.

The co-existing dysenteric disease utterly forbad any thought of amputation. In one instance,—" certainly a very uncommon, and, if not an unprecedented, at least an unrecorded, one"—the effect of the miasmal poisoning was, apparently, the production of *spontaneous dry mortification of both hands.* The patient, a seaman, 20 years of age and previously in excellent health, had suffered from ague and flux in the Yang-tse-Kiang. Soon after arriving at Chusan in October, the affection of the fingers began with aching pain in them, especially at night, preventing sleep, and afterwards with increased suffering; the affected parts were at first dark-coloured, and then black and lifeless. He was received on board the Minden about a month after the earliest appearance of the symptoms. At this time, " the disease had reached the distal joints of the thumbs and all the fingers, but had extended to the second joint of the index fingers, to that extent the circulation had entirely ceased, the parts being dead and black. Complains of acute pain in the hands, shooting up the arms in the line of the ulnar nerves, and becoming much more severe at night;—pulse 108, sharp and irritative, skin dry, tongue healthy, bowels at present act pretty regularly, little appetite, no line of separation between the dead and living parts. The gangrene did not advance after he was received into hospital; the line of arrestation became evident a few days subsequently; separation of the sphacelated extremities, principally natural, but partly artificial, including the removal of projecting portions of bone, proceeded favourably; granulation and cicatrization followed; and, on the 26th, the cure of the stumps being nearly complete, and the general health restored, as his ship was on the point of sailing, he was discharged."

The patient had been treated with full doses of quinine, anodynes, and with occasional remedies for intestinal disorder, which recurred more than once during the course of treatment. In many respects, this case resembled one of *gangræna senilis,* or that form of mortification which arises from excessive cold. Dr. Wilson does not hesitate to regard this curious local affection as the result—an uncommon one, it must be acknowledged —of malarious poisoning.

As we have already said, intermittent fever and dysentery were the prevailing diseases at Chusan. Cholera was of rare occurrence. No case had been observed among the fleet anchored there, till the 4th of November on board the Thalia. The case proved rapidly (in five hours) fatal; the patient had been in perfect health before the attack. Dr. Wilson appears to be a decided non-contagionist; for he says :—

" As yet, it is a solitary case among the ships of war, though there have been some cases among the Indian troops quartered near the town, and the merchant seamen in the inner harbour. The subject in this case was one of a party from the ship, employed for three days previously about a junk, which had sunk with copper on board, close to the shore of the inner harbour, and which they, with considerable labour, partly under water, had been getting up and landing. From that shore, the cause of the disease, whatever it may be, was, there is every reason to believe, in this case, derived; it was one of great intensity, and not destitute of useful information, for it goes to prove the purely terrestrial origin of cholera, its incommunicability by person, and the power of accidental agency—predisposing causes—in its production. The place where the party was employed possesses all the visible qualities of a malarious soil; the patient died in the ship among his messmates where there was no restraint, and no other person was affected by the disease, and all the other men employed in the same duty have heretofore at least escaped it." P. 43.

A week subsequently, another case occurred on board the same ship; but it was much less severe, and the patient recovered.

"The disease has not appeared in any other ship of the squadron, and it should be observed that no other is anchored so near to the plain of Tinghae and the inner harbour. Cases occasionally occur still in the merchant ships stationed there, as well as among the troops; but neither the number, nor proportion, nor the proportion of resulting mortality, is known, as there are no official returns, and the pressure of other duties prevents personal inquiry to any extent." P. 45.

While the squadron had been in the Yang-tse-Kiang off Nankin, there appear to have been about 90 cases, in all, of Asiatic or malignant cholera; of these, 30 had proved fatal. But by far the most serious scourges were agues and the flux. The crews of all the vessels suffered more or less severely from these endemic pestilences; and sad was the havoc among some of them. There was, however, one ship in the squadron, the Apollo, which was so much more healthy during the whole time that she was in the Chinese seas than all the rest, that we are naturally led to enquire as to the probable cause of her superior immunity from disease. This frigate was in the Yang-tse-Kiang during all the operations off Nankin; remained there till the signature of the treaty, and then stayed at Chusan for three months before returning to England. During the whole of this period, she was healthy, absolutely and relatively. Upon communicating with her captain and surgeon, our author found that the only peculiarity in the hygienic arrangements on board the Apollo was, that the lower parts of the ship were regularly washed and purified by pumping water in and out every day in very hot weather, and three or four times a week, when the temperature was low. By this means, though the space under the limber boards was never absolutely dry, no impurities arising from leakage or otherwise were ever allowed to accumulate and give rise to the offensive effluvia of bilge-water. It is a proverbial saying among nautical men, that "a leaky ship is a healthy ship." Whether this be true or not, the prevailing opinion among the medical officers of the navy for many years past has been, that it is an important thing for the health of the crew, that the lower parts of a ship, as well as the inhabited decks, be kept as dry as possible. No one is likely to question the truth of this remark as far as the decks are concerned; but it is very different as respects all the parts under the limber boards; and for this very simple reason, that it is scarcely possible, by any means, to keep them absolutely dry. Now, if this cannot be done, the consequence must be, that there is always under the flooring a quantity of confined and stagnant fluid, which is constantly exhaling offensive and necessarily deleterious effluvia. Such then being the case, it is certainly not unreasonable to believe that it would be better to wash out all the lower parts well and frequently than to leave them in this condition. The frequent admission and expulsion of water will have the effect not only of preventing such an accumulation, but, it may be also, of absorbing and carrying off the impure air, as it is formed, that is necessarily generated wherever there is any decaying matter present; and that such a generation is continually going on in some vessels cannot well be doubted.

Dr. Wilson tells us that, when he was in the West Indies, about sixteen years ago, it was then the usual practice, upon the arrival of a ship on that station, especially if there was any appearance of fever on board, to take out all her stores, and whitewash and dry (by means of stoves) the whole interior, from the hatches to the keel, till all sign of dirtiness or dampness was removed. But what was the result of all this labour, according to his experience? "It happened frequently—more frequently than otherwise—that fever, if it had begun, instead of being arrested or moderated, became more frequent and severe after than it had been before; and often set in at no distant period, if it had not shown itself previously. This relationship the writer pointed out, and offered some remarks on, both in the 'Memoirs of West Indian Fevers,' and the 'Statistical Report on the Health of the Navy,' in the West Indies, especially the former. He was satisfied then, as he still is, that the process in question did not answer the laudable end for which it was ordered, that, namely, of staying or retarding the march of the destructive disease; nay, that it often precipitated the eruption of the disease, and rendered it more violent."

We were certainly not prepared for such a result as this; nor can we well perceive how the thorough purification—by the dry or wet process—of a ship's interior (and this too, be it remembered, in harbour, where every possible facility is procurable) should ever promote the development or spread of any fever. Whether, indeed, the regular and frequent washing out of the hold by the free admission and forcible expulsion of water might not be advantageously substituted for the operation of dry cleansing is a subject that well merits the attention of all naval surgeons, and we trust that they will not fail to test the value of the practice in different climates. Our author is evidently inclined to think favourably of it; and his opinion is deservedly entitled to much consideration. He alludes to the late very lamentable case of the Eclair (recently returned from the coast of Africa), and suggests that the process noticed above might perhaps have been employed with benefit.

The fearful pestilence on board this steamer was, he thinks, derived, in a great measure at least, from the ship herself.

"Every thing in the progress, character, and cessation of the disease in the Eclair, so far as the writer has the means of judging, points to its local origin, and shows that it was generated on board, its cause being the product of chemical action within the vessel, probably in her lower and least accessible parts. It has been alleged that the disease was propagated by personal contagion. This is not the place to discuss the subject, but it may be asked in passing, where are the proofs of the allegation? The great cumulative argument, and the only one which can satisfy rational scepticism, *that, namely, of its extension to healthy persons living among diseased persons, after the latter were removed from the place where they sickened, is altogether wanting.* Had any one person attending on or communicating with Dr. Rogers and the other fever patients, after they were moved from the Eclair to a sound ship, been affected, the person so affected, not having been on board the Eclair, the case would assume an entirely different aspect." P. 93.

With the removal of the affected from the Eclair, and the termination of fever in them, the disease entirely ceased.

What has now been said in reference to the (probable) good effects of frequent lavation of the interior of a ship's hold, may be applied with equal force to the lavation of the ship's company, by the regular and, when possible, the daily use of sea-bathing, or ablution with salt water. Sailors have, in this respect, a great advantage over soldiers, who are so often not only quartered in an unhealthy district, but have few or no facilities of personal ablution and refreshment.

The Minden remained at Chusan until May 1843, and then sailed for Amoy, on her way to Hong-Kong. Perhaps a few remarks here on some of the more prominent features of Chusan and its people may not be unacceptable to our readers.

The island is described as being highly productive. Every spot of ground, from the sea-beach to the summits of the hills, is under cultivation. Rice is the principal growth; but, besides this grain, millet, maize and wheat, are also grown. There is abundance too of excellent vegetables. There are very few forest trees to be seen. Bamboo is, in the arts of life, what rice is in respect of food—a substance of almost universal application.

The Chinese seem to have anticipated, in practice at least, some of the recent suggestions of scientific agriculturalists on the highly important, albeit somewhat unsavoury, subject of manure. Human ordure is everywhere most carefully collected and preserved; it is valued to a degree which would seem perfectly ridiculous in other places.

"Close to the houses, which in the country are grouped into hamlets in the hollows, there are placed large earthern pans, into which are thrown all fragments of decomposable substances, to which are added the desired quantity of fluid, including urine. With heat varying from 80° to 86° in summer, destructive fermentation proceeds rapidly, and multitudes of maggots are developed, giving life and motion to the loathsome compound. Scarce anything can be conceived more disgusting than the contents of these vessels; but the Chinese disregard their offensive qualities, looking to their useful ones. They arrange them in rows along the public paths, and always place them near, if not in contact with, their houses, that they may at once commit to their keeping every particle of available matter. When putrefaction has proceeded to the point of giving the highest degree of fertilizing power to the mass, it is transferred to buckets, and carried to the fields, often to considerable distances, and when the ground is steep, with great expenditure of human strength; for, except when yoked to the plough or harrow, man's labour is not lightened by ox, horse, or other quadruped. Placing a bamboo across the shoulders, with a bucket at each end, they carry what they consider, and what no doubt is to them, very precious stuff, in large quantities, to distant places, and often difficult of access; showing at once the value of productive soil, the industrial habits of the people, and the muscular power of the persons who perform this part of the farm labour. These receptacles of filth, and sources of fertility, are not confined to the country; the towns have an abundant share of them." P. 12.

The cold in the winter season at Chusan must be severe; for we are told that the people store up immense quantities of ice, chiefly for the purpose of preserving fish, which forms a large portion of their food in summer.

The chief diseases of the natives appear to be the same as those which have affected our troops quartered in the island, and the crews of our ships anchored off it. These, as already mentioned, are remittent and intermittent fevers, and bronchial and intestinal irritation, in the form of catarrh, diarrhœa, dysentery, and so forth. Cholera has occasionally prevailed to a great extent, and occasioned severe mortality. The principal external affections seem to be scrofula, ophthalmia, and various cutaneous maladies, including elephantiasis. Scabies is disgustingly common; nor are much pains taken to get rid of it: even a Mandarin feels no shame in shewing his hands—and this at the dinner-table too—covered with the itch!

The food of the working-classes consists almost exclusively of vegetables, especially rice, with salted fish. The immoderate use of tea, and of opium and tobacco-smoking, must tend to deteriorate the constitution. The opium is used as a luxury; the tobacco has become almost a necessary of life: "they never drop the tobacco-pipe; smoking from morning to night, and drinking largely, at short intervals, a miserably weak infusion of coarse tea." Their habits in reference to ablution, are intolerably filthy; they seldom or never change their garments. The following remarks on Elephantiasis may be read with interest.

"Elephantiasis, judging from the number of cases casually seen, is as common here as at Rio de Janeiro, or even at Barbados, where it is so prevalent as to have given rise to one of its names—*Barbados-Leg.* It is generally, and there is reason to believe justly, considered as an endemic disease in the proper meaning of the word. Yet, on examining the topographical and appreciable climatorial constitution of the three places, few points of agreement can be discovered between them, though in some peculiarity of these things in conjunction with the modes of living and personal practices, the causes of endemic disease must consist. In the latter particulars, it is true, in diet and domestic management, there is considerable similarity between some classes of persons, in the positions named. Both at Rio de Janeiro and Barbados, elephantiasis prevails principally among the negroes, who, like the poorer Chinese, subsist almost exclusively on vegetables, with a portion of salted fish. Like them, they are also very often dirty in their persons, their cabins, and their clothes. These things may have much influence in producing the disease, but it is evident that they are not of themselves sufficient; for other classes and races of men, who feed as poorly, and are not more cleanly, are not affected by it.

"Respecting the nature and rational treatment of elephantiasis, there is fortunately less doubt than on the subject of its origin. It consists essentially in inflammation of the subcutaneous cellular tissue, which leads to effusion, the effused matter becoming imperfectly organised, occasioning tumefaction, and impairing more or less the motive power of the limb. Repeated attacks of inflammation, effusion, and consolidation, produce, if not arrested, in the course of time, generally many years, the highly enlarged, tuberculated, scaly leg, resembling very closely that of an elephant. As the disease advances, the functions of every tissue, especially of the skin, ligaments, and bones, are injured or destroyed, by increasing pressure, till, beginning with the separation of the toes, disorganization proceeds to a fatal issue." P. 25.

By far the most efficient local remedy is the copious abstraction of blood by incision or free puncture of the diseased integuments. The genital organs are, it is well known, a common seat of Elephantiasis. Besides the diseases already mentioned, Small-pox is not uncommon: Syphilis too is frequent in the sea-port towns.

When the Minden arrived at Hong-Kong in the beginning of June, remittent (not intermittent, as at Chusan) Fevers and Dysentery were found to be very prevalent, Very generally these two morbid states were associated together; so that it was often difficult to say which was the primary and principal disease. "Whichever appeared first, it constantly happened that, as one series of morbid actions declined, the other rose." This circumstance added greatly to the difficulty experienced in the treatment of the patients; for what promised relief for the one state, was anything but advisable for the other. As the severity of the fever declined, it often, nay generally, assumed an intermittent type; and very frequently when this was subdued, gastric irritation with flux set in, to be succeeded perhaps by the recrudescence of the periodic fever.

In most cases of the Hong-Kong remittent fever, the cerebral symptoms were strongly marked; hence it was usually called "head fever" among the sailors. In the worst cases, the febrific miasm acted like a concentrated poison, paralyzing all the powers of life. In some instances the symptoms, at the outset, resembled a good deal those of malignant cholera. Dr. W. scouts the Broussaian idea of the disease being the result of any local inflammation: it is truly and strictly an essential fever. Dissection threw but little, if any, light, upon its nature: congestion, not inflammation, was the prevailing character of the morbid appearances observed in the viscera.

Sloughing ulcer was a much more rare affection at Hong-Kong than it had been at Chusan. This, and the circumstance of remittent taking the place of intermittent fever, were the chief points of difference in the medical constitutions of these two localities. In the dysentery of Hong-Kong, the stomach and small intestines were more frequently affected than in the same disease at Chusan.

On the whole, Hong-Kong seems to be anything but a healthy spot. The troops have suffered fearfully from the effects of miasmal poison—arising, no doubt, from the swampy ground in the neighbourhood of the new town Victoria, which used to be cultivated for rice plantations. Dr. Wilson remarks more than once that those persons, who resided in the central parts of the town, suffered much less from fever and dysentery than those living in the outskirts. He strongly recommends that the cultivation of rice (which, it is well known, always requires a great deal of moisture) should be entirely abandoned, and that of wheat and other cereal grains, be substituted. But it should always be borne in mind that, unless the latter part of this recommendation be carried into effect, no change at all should be made; as "a cultivated rice-field is much less dangerous than the broken, swampy, weed-covered condition of the surface which follows its neglect." It is therefore a most important point for all settlers in a place like Hong-Kong to attend to this circumstance.

We shall now direct the attention of our readers to the most practical parts in the present volume—those which describe the treatment of the diseases which constituted the great bulk of the cases received on board the hospital ship, while stationed at the two ports in the Chinese seas,

Chusan and Hong-Kong: and first, of the *Remittent Fever*, endemic at the latter place.

In the worst cases of this fever, characterised by extreme depression, the first and most prominent indication was to sustain the sinking energies of life by the use of stimulants and cordials. When reaction followed, the principal remedies were local abstraction of blood, blisters, occasional aperients, effervescing draughts, and moderate doses of calomel with or without opium, and sometimes with ipecacuan. In a few cases, more active depletion was necessary; but this was rare: in some cases, the detraction of blood was clearly injurious. The chief danger consisted in the marked tendency to excessive debility and fatal collapse; and, as this was apt to supervene even after the stage of reaction had set in, the greatest watchfulness was required on the part of the medical attendant. Hence "the practice was often one of expedients, and was based less on fixed principles than the pride of science is willing to admit." Dr. Wilson is very earnest in deprecating the use of large bloodletting, merely because the pyrexial symptoms may run high during the exacerbation of the fever. The local abstraction of blood is almost always much safer, and is, moreover, generally sufficient. The doses, in which Calomel was given, were either one grain every two hours, or three, four, or five grains two or three times in the 24 hours. This was considered a safer practice, and better suited for the ends aimed at, than scruple or half-drachm doses two or three times daily. Dr. W. candidly confesses that, on the whole, the results of the calomel treatment were not very flattering to the practitioner's hopes. There was reason to fear that the appalling collapse and rapid sinking were sometimes rather accelerated than otherwise by the unguarded use of this energetic medicine.

When the remissions became more decided, and the exacerbations less violent, the chief reliance was placed upon quinine; but our author expressly says that, "although it sometimes answered expectation, it more frequently disappointed. Here, as in intermittent fever, neither it, nor any other form of cinchona, could in many instances be long tolerated, being speedily followed by, if it did not excite, *flux*."

We must not close our remarks on the treatment of Remittent Fever without mentioning that Dr. Wilson has strongly insisted, at the beginning of his work, on the great importance of Emetics exhibited at the outset of most febrile affections, including Cholera and choleroid attacks. He very justly remarks, that "the practitioners of the last age made much more use of emetics than those of the present; they might employ them without due discrimination, and therefore empirically in some instances; and they might entertain erroneous notion as to the grounds on which they were administered: but, however these things might have been, it may be affirmed that emetics do not, at present, hold the rank they are entitled to among therapeutic agents; and that, especially at the onset of many febrile diseases, they are undeservedly and injuriously neglected."

*Intermittent Fevers.*—The most frequent type was the quotidian, and then the tertian: quartans did occur, but not often. We need scarcely say that the treatment consisted in the exhibition—premising a warm bath, and occasionally a brisk purge—of quinine; two grains in solution every three or four hours during the intermission. Under this plan, "success was sure, and generally speedy," even when the disease had continued for a length of time. (The cure was never considered complete till some weeks had elapsed after the last accession, however slight, of the fever.) This remark, however, applies only to those cases in which the fever was uncomplicated. Whenever there were other forms of disease, more especially Dysentery, co-existent, the greatest difficulty was experienced in effecting a cure. The very existence of the febrile disease was often masked by the intestinal affection.

" Sometimes it was, for a while, so much disguised as to require close observation and questioning to detect its existence; and when its recurrence was evident, there was often regularity in the time of accession, as well as in the duration, and development of the different parts of the paroxysm. Rigor was seldom well-marked; sometimes the hot stage was wanting, the paroxysm consisting chiefly in profuse sweat, with sensations of chilliness rather than heat; instead of being ushered in by coldness and shaking, the invasion was occasionally characterised by numbness or total temporary suspension of sensibility in the lower extremities; and there were other anomalies and discrepancies of other kinds in the febrile paroxysm, difficult to describe, and unnecessary to detail, which were yet of such a nature as to leave no doubt of their being products of the miasmal poison and the integral parts of the periodic fever." P. 204.

The intestinal irritation prevented the exhibition of *the* remedy for the ague. Neither quinine, nor any other form of cinchona, could be borne; and even simple bitter infusions often seemed to disagree with the patients, after the flux had been arrested. Under these circumstances, a trial was made of *arsenic*. The following practical remarks on this potent remedy deserve notice.

" In many instances, it failed to subdue the disease or lessen its violence, but in others, which, without its intervention, seemed hopeless, its remedial efficacy was unquestionable. When there was neither destructive organic lesions nor helpless exhaustion, conditions alike irremediable by any means, its power over the periodic paroxysm soon became apparent: and, when fully established, generally continued till their recurrence was finally prevented, and the febrile part of the combined affection was consequently cured. While acting thus favourably on the fever, it did not, as was apprehended, aggravate the flux; nay, it seemed, though the appearance was not so clear as to warrant a positive conclusion, to produce a beneficial effect on it also. At any rate, while it did not injure, it did not prevent the use of such means as might prove directly curative in the alvine affection." P. 205.

The dose, usually employed, was three or four minims of the solution in camphorated mixture, or infusion of Diosma or Buchu leaves, (which acts serviceably in some forms of flux,) two or three times daily.

*Acute Dysentery.*—In the early stage, when there is high fever, and the abdominal symptoms are severe, a large bloodletting—from 25 to 30 ozs., if faintness does not occur—followed by a warm, or rather a hot (105°) bath, is the remedy on which we must chiefly rely.

" Within a few hours—six will be sufficient to show the efficacy of what has been done—if there be not the most unequivocal yielding of all the urgent symptoms, recourse must be had to local bloodletting. From fifteen to twenty-five leeches should be applied to the abdomen, and ten or twelve round the margin of the anus; a moderate quantity may often be drawn from the margin of the false ribs, about the same time, by the cupping instruments. On the completion of these measures, the bath may again be employed; after which, the patient being dried, and speedily carried to bed between blankets, the abdomen should be covered with a light warm poultice. Twelve hours hence, or twenty-four hours after admission, if there be any doubt as to the disease being mastered, and yielding rapidly, a large blister must be laid on the abdomen, not a patch covering a hand-breadth, but a sheet extending from the pit of the stomach to the pubes, and from one iliac hollow to the other." P. 208.

Calomel and opium—to which, in most instances, ipecacuan or antimony (?) will prove a useful addition—are the internal remedies which, in our author's opinion, deserve the chief reliance. The average doses may be stated at four grains of calomel, one grain and a half of crude opium, and the same quantity of ipecacuan, to be given every third hour. The patient should not be teased with any other medicine. By the use of the means, internal and external, now mentioned, "nine cases in ten of the form of dysentery under consideration, at the stage indicated, will be successfully treated."

Dr. Wilson does not approve of the use of purgatives in acute idiopathic dysentery. "Retained secretions," he quaintly remarks, "like pent-up fæces, have a larger place in the imagination of the practitioner than in the person of the patient."* The theory, as well as the practice, of Dr. Wilson is much more likely to find favour and followers than the strange doctrines—would that they were merely so—of Dr. MacGregor, as described in the last number of this Journal. With respect to the *modus operandi* of mercury—in conjunction with opium—in dysentery, our author is of opinion that it acts, not by exercising any specific power over the intestinal affection, or any peculiar influence on the liver, as some writers have supposed, but solely as a general sedative and antiphlogistic. Hence its marked utility, not in dysentery only, but also in all febrile affections in which any part or organ of the body is "in such a state of vascular derangement, inflammatory or congestive, as to threaten rupture of vessels, abscess, ulcer, gangrene, or other form of disorganization." And here we may remark, that it is a great, although far from being an uncommon, fallacy to suppose that the mercury must be pushed to the extent of salivation, if we hope to effect a cure of dysentery and some other diseases. It is surely enough—we are appropriating the words of our author—that the morbid condition is removed, whether one of the ordinary, certainly not curative, effects of the medicine be realised or not. Not to be satisfied with this point argues a want of common sense, not to talk of professional reflection.

---

* This allusion to retained secretions, &c., suggests to our mind that it might perhaps be well if medical men were in the habit of examining the more obvious chemical properties—we allude more particularly to those of acidity and alkalinity—of the thin alvine evacuations in a disease like acute dysentery. That there is sometimes an inordinate excess of acid in the black vomit of Yellow Fever is a fact that has, we believe, been distinctly proved, and to which reference was made in a late number of this Journal, No. 85, p. 281.

Whether the association of mild alkaline medicines along with mercurials and opium might not be beneficial, is a question not undeserving of attention. Does not chalk act as an astringent, in most cases, by neutralising a predominating acid in the intestines?

But mercury is not always remedial in dysentery, even when administered with judgment and in moderation. In some cases, the system will be found to be intolerant of its action; it produces great depression, and, if persevered with, fatal collapse. The treatment of such cases requires the greatest watchfulness and discrimination. As a matter of course, the use of the mercury must at once be suspended, or altogether given up.

When the Dysentery is chronic, astringent enemata—zinci sulphat. gr. x., aquæ ℥vj., tinct. opii ʒss.—have been found extremely useful in allaying the irritation of the bowels and checking the intestinal discharges. The exhibition of mild aperients, as magnesia and rhubarb, castor oil with a few drops of laudanum, is often beneficial in this stage of the disease.

The Dysentery, that prevailed on the coast of China, was unusually intractable; and for this reason, that it was seldom an idiopathic uncomplicated affection; but most frequently associated with Intermittent Fever, secondary to, and alternating or coexisting with it. In many cases it was associated with ulcers of the extremities; " generally as one form of disease rose in force, the other fell." These complications cease to surprise us, when we bear in mind that the three morbid actions were doubtless attributable to one and the same cause, viz. miasmal exhalation.

The febrile excitement in the first stage was seldom so great as it usually is; nor were the patients able to bear the same amount of depletion. The doses of calomel, opium, and ipecacuan required to be considerably diminished. Salivation was carefully avoided; the exhaustion, consequent thereon, was too great for the patient's strength. Dr. Wilson believes that many lives have been sacrificed to the injudicious administration of mercurial medicines.

" In a few cases, when the dejections consisted chiefly of blood, acetate of Lead produced good effects:" to such cases, its beneficial influence was confined. The sulphate of Zinc did not maintain its reputation in any form of the disease. The nitrate of Silver was not more successful. With respect to astringents generally (in the chronic disease), our author makes the important practical remark, that none of them " had the power of restraining the discharges even temporarily, in most cases; and, when that effect was produced for a little, it was constantly accompanied by more severe tormina, and soon followed by increased frequency of stools."

The application of a few leeches to tender parts of the abdomen, or round the anus, always produced relief. Effervescing draughts with a few drops of opium or hydrocyanic acid were most grateful, and, often too, very useful. In lieu of blisters to the abdomen, the following rubefacient was employed with good effect.

" An ointment, consisting of equal parts of mercurial and iodine ointment, with a small portion of cantharides plaster, was rubbed carefully over the abdomen, to the extent of a drachm daily; after which, a flannel roller was applied. In this way, the irritation of a large blistered surface was avoided—no light consideration, when there was tendency to sinking; and the derivative action thus established, without considering any other, was not only more steady and constant, but also more effectual on the whole. If vesication, in a slight degree, took place, the application was suspended, to be resumed if required." P. 220.

In a great number of cases, the disease resisted every variety of medicine and mode of treatment. We have already mentioned that the infusion of the Diosma Crenata or *Buchu* seemed to answer, on the whole, better than any other vegetable tonic in giving strength to the bowels, in many of the forms of chronic flux. During the progress of convalescence, chalybeate preparations were occasionally of marked benefit: the *tinctura sesquichloridi*, in full doses, was preferred. Under its use, the complexion often improved, and strength was gained. In all cases, the food that was allowed was given in small quantities at a time and of the simplest nature: "moderate quantities of farinaceous food were (often) alone compatible with a curative process."

With respect to the Dropsical effusions that so frequently occur in the course of chronic dysentery, it will be best to give Dr. Wilson's observations in his own words.

"In all such cases, the principal object aimed at was remedy of the condition on which the dropsy depended, comparatively little attention being paid to the effusive effect. That condition consisted almost universally in organic weakness; organic obstructions, considering the nature of the primary disease; and its powers, real and imputed, of producing structural lesion in other places, were singularly rare. Little was expected from diuretics, and they accomplished even less than was looked for. To specify the various means resorted to in the dropsical complications, would be little more than a repetition of the measures used in periodic fever and flux. Irregular ague, frequent purging, ascites, anasarca, and extreme debility, co-existent, presented as hopeless a subject for medical management as can well be conceived. Of such cases, there were many, most of which proved altogether intractable; but, in some of them, decided advantage, more certainly than from anything else, was derived from the combination of arsenic and buchu.

"In a few cases where effusion was confined to the abdomen, anasarca having ceased, and other cavities being clear, friction with the compound ointment, already twice referred to, was followed by marked improvement. Under its use, tumidity subsided, sometimes permanently. Whatever may be thought of the physiological fitness of the application in this or the other affections, no doubt is entertained of its practical utility." P. 225.

Perhaps this will be as good a place as any to mention that there was an unusual tendency to the generation of Intestinal Worms among the sick in China. Dr. W. remarks upon this subject:—

"There is great disposition to the formation of intestinal worms, almost exclusively lumbrici, here (Hong-Kong), as well as at Chusan, which are sometimes generated in extraordinary numbers, being occasionally voided by the mouth, but more commonly by the anus, and giving no symptoms of their existence till they are discharged: they are found in masses on post-mortem examinations, after fever and flux; the former, as frequently as the latter, when protracted. Their extensive prodution has been ascribed, erroneously it is believed, at Chusan, to the water found there, containing, as it sometimes does, portions of earthy and vegetable matter; for they are as numerous at Hong-Kong, where the water is singularly clear, and free from admixture of any kind, except small quantities of mineral substances, which it holds in perfect solution, and which cannot be supposed conducive to such effects. There is little apparent difficulty in accounting for the abundance of these parasites in China; it can scarcely be questioned that the excessive tendency to, and occasional accumulation of, them, arises out of the enfeebled, unhealthy condition of the alimentary apparatus, more particularly of the interior membrane; being infested by depraved secretions, and coated with adhesive mucus, it ceases to perform it proper functions adequately; and from the same cause, becomes the prolific bed of those creatures. Neither their origin in the intestines, mode of production, nor introduction, is of any practical importance; it is probable that their ova or elementary germs are extensively diffused, and constantly carried into the stomach with dietetic substances: it is certain that their existence is incompatible with a sound state of the living parts, in which they are developed; and that what is required for their prevention is vigorous healthy action in these parts." P. 193.

*Sloughing Ulcer.*—We have already remarked that there was a marked tendency to this very troublesome form of disease on board the Minden in her outward voyage. *Then,* it was believed to arise from the operation of a peculiar miasm evolved from the hold and lower parts of the ship; and that this was the cause is rendered highly probable by the circumstance that, when all the stores were taken out at Chusan and the vessel well cleaned out, no new cases occurred for a considerable time. The morbid tendency, however, became afterwards re-developed, in consequence of the operation of malarious miasms; for the connection between the ulcerative disease, and the periodic fevers that prevailed, was too obvious to escape the notice of any one. They doubtless arise from the same morbific influence, and are but different indications or exponents of its operation upon the system.

By far the most effectual remedy in the *treatment* of the sloughing ulcer was the employment of free incisions in the surrounding integuments.

"In a great majority of cases, the sloughing process had advanced far before the patients were received; in some, as formerly stated, bones were denuded, and tendons and ligaments destroyed. But in very few, even where the destruction was greatest, was treatment by incision omitted; and in those only where, from fever or flux, there was great constitutional debility. The amount of incision was regulated by the extent of disease in the tissues under and around the ulcer. In some instances, where it did not descend below the integuments, the ulcerative process being phagedenic rather than gangrenous, and the destruction neither very rapid, nor reaching under tissues, it was sufficient to relieve the more superficial vessels, and to substitute scarification for what is understood by incision. More frequently, however, it was necessary to use the knife freely, passing it through the skin, and into the underlying cellular structure. Whatever the proper depth might be, the scalpel was carried quickly from beyond the limit of surrounding disease to the ulcer, often through it. The distance between the incisions varied, but was generally less than a quarter of an inch; their direction was most frequently parallel in the line of the limb, occasionally radiated from a circle, clear of the affected integuments, to the ulcerated centre, according to the position of parts, and degree of vascular action. In many cases, it was necessary to repeat the practice; in some, frequently." P. 227.

The effects of this mode of treatment were generally prompt and most satisfactory. The relief to pain and irritation was often immediate; and, although the remedy was a painful one, patients sought rather than shunned its repetition, upon the recurrence of bad symptoms; so unequivocal was the benefit derived from it.

"Instead of sanious fœtid discharges from the ulcer—its ashy, livid, or black surface, and abrupt margin—there was secretion of pus, separation of sloughing matter, and a crop of florid healthy granulations; the surrounding parts, which had been tumid, darkly inflamed, or œdematous, or having both conditions combined, became flaccid and shrunken, assuming the pale complexion of health. In no instance did the sloughing action extend to the incised surfaces, which either healed speedily by adhesion, or more slowly, but not less surely, by granulation. This, which may appear strange, is unquestionable, and an important fact; for if there were any doubt respecting it, the value of the operation would be lessened, if not rendered problematical." P. 228.

But it was not only in the Sloughing Ulcer that so much benefit was obtained from the treatment now recommended. In many cases of Indolent Ulcer—with flabby granulations, and a thick, hard, cartilage-like margin—" nothing availed so much as carrying a scalpel from the periphery of the diseased tissues to the centre, cutting freely, and at many points, through the indurated ring which formed the limit, and prevented the extension of cicatrization." In some cases, the practice required to be repeated several times.

To promote the flow of blood, and to keep up a discharge of sero-lymphous fluid from the incisions, water dressings, warm or tepid, were always employed for two or three days, or longer if deemed necessary. When the sloughing process had ceased, and the ulcer had put on the appearance of healing, the solution of Chloride of Lime and diluted Nitric Acid were found useful, by gently stimulating the part to more vigorous action. " In some cases, where there was unhealthy, phagedenic or partially sloughing, action, the parts beyond the ulcer being sound or little affected, undiluted nitric acid was very useful; and in others, especially those marked by œdema and other symptoms of inaction in the surrounding surface, a solution of nitrate of silver more frequently the rod itself, was applied with excellent effect, to the diseased integuments, as well as to the ulcer."

After the ulcer had healed, or nearly so, much benefit was often derived from the daily affusion of cold sea-water upon the limb. Considering the constitutional origin of the form of Ulceration now under notice, and its intimate association with Ague and Dysentery, we need scarcely say that appropriate internal treatment was invariably employed along with the use of those external remedies which we have already described.

The results of the treatment were, upon the whole, very satisfactory, as appears from the subjoined statement:—

" One case sketched in a former page, both lower extremities being affected, with great complication and resulting debility, terminated fatally. In another instance, it was necessary, on account of extensive, progressive, and hopeless disorganisation, to remove a limb below the knee. But all the other cases ultimately terminated in recovery. From the amount of destruction, the restorative process was protracted in most; it was uncertain and vacillating in many, frequently arrested in some, the condition being all but desperate, and giving rise to grave questions about the duty of amputation. In such a state of things, it is much to escape with life, and entire limbs; and, although some of the subjects may never be fit for very active duties, on account of the extent and position of cicatrized parts, the effects of the measures instituted, and most zealously, as well as skilfully carried out, are felt to be not only satisfactory but gratifying." P. 232.

The hot season—from May to October inclusive—of 1844 was altogether much less sickly than the corresponding period in the preceding year. It is often, nay generally, impossible to account for these changes in the " medical constitutions " of different seasons: for, as far as we yet know, there is no steady relation between the condition of a season in point of salubrity, and any barometric, thermometric, hygrometric, or other appreciable states of the atmosphere. Although, however, we cannot explain the cause of such hygienic differences, while the physical conditions of the air and earth are seemingly very much the same, the truth of the fact cannot be disputed; and due attention to it, it should ever be remembered, is of great importance in estimating the value of any remedies or modes of treatment which may be recommended, at any time, from their alleged superior efficacy. A line of practice, that may suit the epidemic of one year or of one place, may be utterly valueless or even injurious in that of another. Hence the fallacy of laying down any dogmatic rules as to the treatment of an epidemic or endemic disease, without reference to the "medical constitution" of the particular season or locality; and, at the same time, the danger of building any structure of therapeutic principles on the results obtained by Mr. this and Dr. that, in such a year or such a place. Dr. Wilson very justly remarks, that "a practitioner arriving here (Hong-Kong) in the summer of 1844, would find a much larger proportion of the sick under his care recover, than did the practitioner of 1843; he would, probably, with a laudable desire to accomplish more than the men earlier in the field had done, modify the means of treatment used by them, or apply something different: and to this modification or alteration of means, not to the comparatively slight morbid impression, he would be apt to ascribe his better fortune, counting it the effect of professional merit, not of changed circumstances. Having satisfied himself of this, he, and some others who are prone to believe much and to hope much, readily arrive at the conclusion, that his predecessors were inefficient practitioners; that they did not understand, or did not properly perform, what was required of them by the obligations of their office; and that they are, therefore, chargeable with the heavy crime of having allowed men to die who might have been saved."

He sums up with the humiliating, but nevertheless the useful because the candid, admission that

"The more that is seen of some forms of disease, and the more closely they are studied, including especially the precipitous fevers of the tropics, the more likely is the conviction to come and deepen, that medicine has often but little remedial control over them; and grave questions will then arise as to whether artificial appliances, confused and contradictory as they often are, may not prove injurious rather than beneficial—if not curative or tending to cure, will they not become instruments of harm? Experience also shows that, while we think we are making rapid onward progress, we are often moving in a circle only, returning, after a long, toilsome, unprofitable journey, to the spot where our ancestors arrived long ago. This truth, which should humble and correct, not arrest, us in the course of diligent sober inquiry, meets us at every turn. One example out of many may be alluded to, namely, the treatment of dysentery in India, though that of fevers in general would not be less in point." P. 190.

In short, more benefit is, in many cases at least, to be looked for in the direction of Hygienic and preventive, than of Therapeutic or curative, Medicine. The great improvements that have been effected in preserving the health of our sailors, more especially by the superior arrangements in reference to their food and clothing, give ample encouragement for the reasonable expectation of still further ameliorations in the way of protection from disease, by the diligent application of different hygienic measures to the varying circumstances in which our soldiers and sailors may be placed. We have seen the strikingly good effects of a particular practice on board one ship—the Apollo. Why then may we not expect that other plans and methods of hygienic improvement may be discovered? The subject is one of first-rate importance, and should be never out of the mind or thoughts of naval and military surgeons while engaged in those climates where fevers, dysentery, and such like diseases are always more or less prevalent.

Dr. W.'s last Chapter gives us a brief, and necessarily a very imperfect, sketch of Chinese medicine. As yet we know too little of the people to be able to appreciate, with any degree of fairness, the progress which they have made in the healing art. From our author's account, it would seem to be in the most rude and contemptible state. In medicine, as in other branches of knowledge, the Chinese appear to have remained for centuries —it may be for tens of centuries—in a state of "petrified fixedness," which nothing has moved. In their physiology, they recognise five elements—fire, water, earth, metal, and wood! They have a vast materia medica, and the physician's prescriptions usually contain not fewer than nine or ten ingredients.

As a matter of course, in a country where anatomy is so utterly neglected, the practice of Surgery must be very rude and ignorant. John Chinaman is not a bold reckless character; and he therefore never goes deeper, in any of his surgical operations, than the surface. A vein is never opened designedly; for there is the greatest horror of shedding blood, except by the hands of the executioner. The moxa is a favourite remedy among the Chinese doctors. Their mode of inoculating for Smallpox—for this practice seems to have been followed in China long before it was dreamed of at Constantinople—is curious :—

"Instead of introducing the virus directly into the system, by a slight incision, they accomplish their object in a circuitous and rather complicated way. The crust of a maturated pock is thoroughly dried, powdered, and rubbed into the mucous membrane of the nostril, or a piece of cotton, powdered with it, is stuffed into the nose. This is the most common method for the common people; but there is one of greater pretension, though probably less effectual, for the rich. A small metallic cup, shaped like the bowl of a tobacco-pipe, is introduced into the nostril of the child, while the inoculator, applying his mouth to the stem, blows the variolous contents forcibly against the lining membrane." P. 247.

The shops of the Chinese druggists seem to be not much unlike those of our own pharmacopolists. They are described as being large and commodiously fitted up with a great array of drawers and jars, arranged much in the same way as in England: glass vessels are rare. Different departments are allotted to separate classes of medicaments; care is taken to keep things in order; and there is a degree of neatness and method in their appearance, which would not be discreditable to a London laboratory. They do not seek notice by party-coloured bottles and cabalistic signs, which make so great a figure in the windows of English medicine venders, but are rigorously plain, and, as far as mere appearance is concerned, appropriate.

With the exception of camphor, rhubarb, and liquorice, there was scarcely anything in the shops that could be recognised. On the counters is usually to be seen a number of boxes containing ready-prepared panaceas for curing cholera, imparting strength and vigour, exciting love, and so forth.

Here we must take leave of our author. His work is very interesting, and contains a great deal of instructive matter. The observations of so experienced a practitioner as Dr. Wilson are well deserving the attention of all medical men, and of our naval and military surgeons more especially. We trust that his example will be followed by some of his brethren in both departments of the Public Service. How full of interest would a medical history of the campaigns of our army in Cabool, for example, be to the profession at large!

After this article had been printed, we accidentally met with the following strongly corroborative testimony by the late distinguished Editor of this Journal, in favour of Dr. Wilson's opinions on the important subject of deleterious miasms often generated from the bilge-water, and other impurities in the hold of a ship. In his review of Sir William Burnett's official report on the destructive fever on board H. M. S. Bann, in 1823 (vide *Medico-Chirurgical Review* for January 1825), Dr. Johnson has remarked:—

"It has now been well ascertained, from fatal experience, that, while we are seeking for external sources of contagion, we have the enemy in our own bosom. Between the timbers and planks in the holds of vessels, miasmata have been generated that scattered destruction among the unsuspecting crews above. The Pyramus was a recent and melancholy example. To this source we are strongly inclined to attribute the fever in the Bann, aided by the atmospheric exposure and intemperance alluded to by the surgeon. It is not a little in favor of this local origin that the fever began forward in the ship, and came gradually to the after part, till nearly all the officers and men were affected."

The Pyramus frigate, alluded to in this extract, suffered dreadfully from fever in the West Indies. After all means had been tried to check its ravages on board, it was determined, at Antigua, to have all her stores and provisions taken out by negroes. Still the pestilence did not entirely cease. At length, the limber-boards in the hold were taken up, when such an offensive smell issued forth, as caused fainting in some on the spot, and several of the officers attending on this duty were immediately seized with the fever.

"The state of the hold, under the limber-boards, is compared by Staff-surgeon Hartle, to a bog of the most pestiferous nature on shore; and Mr. Comrie mentions that three or four of the slaves, employed in cleaning the ship, were attacked with the fever. I may here observe, that previous to the sailing of the Pyramus from England, she had had her magazine fitted on a new plan, and that the shavings and chips resulting therefrom, instead of being removed from the ship, were unfortunately suffered to remain in the lower part of the hold, and thus mixed with the bilge-water under the limber-boards.

"Dr. Fitzgerald states, that sometime previous to the appearance of the fever, the foul state of the hold was sufficiently indicated by smells of a very disagreeable nature issuing therefrom, and diffusing themselves over the ship; and that a candle, when placed at 'the mouth of the hold,' was immediately extinguished; and I was also informed by an officer of the ship whom I formerly knew, and whom I lately saw at the Royal Hospital at Haslar, that all the captains of the hold had lost their lives."

